# A Mechanosensitive Channel, Mouse Transmembrane Channel-Like Protein 1 (mTMC1) Is Translated from a Splice Variant *mTmc1ex1* but Not from the Other Variant *mTmc1ex2*

**DOI:** 10.3390/ijms21186465

**Published:** 2020-09-04

**Authors:** Soichiro Yamaguchi, Maho Hamamura, Ken-ichi Otsuguro

**Affiliations:** 1 Laboratory of Physiology, Department of Basic Veterinary Sciences, Graduate School of Veterinary Medicine, Hokkaido University, Sapporo, Hokkaido 060-0818, Japan; 2 Laboratory of Pharmacology, Department of Basic Veterinary Sciences, Graduate School of Veterinary Medicine, Hokkaido University, Sapporo, Hokkaido 060-0818, Japan; maho.hamamura@gmail.com (M.H.); otsuguro@vetmed.hokudai.ac.jp (K.-i.O.)

**Keywords:** *Tmc1*, mechanosensitive channel, translation, upstream open reading frame, Kozak sequence

## Abstract

Mechanical stimuli caused by sound waves are detected by hair cells in the cochlea through the opening of mechanoelectrical transduction (MET) channels. Transmembrane channel-like protein 1 (TMC1) has been revealed to be the pore-forming component of the MET channel. The two splice variants for mouse *Tmc1* (*mTmc1ex1* and *mTmc1ex2*) were reported to be expressed in the cochlea of infant mice, though only the sequence of *mTmc1ex2* had been deposited in GenBank. However, due to the presence of an upstream open reading frame (uORF) and the absence of a typical Kozak sequence in *mTmc1ex2*, we questioned whether mTMC1 was translated from *mTmc1ex2*. Therefore, in this study, we evaluated which splice variant was protein-coding mRNA. Firstly, the results of RT-PCR and cDNA cloning of *mTmc1* using mRNA isolated from the cochlea of five-week-old mice suggested that more *Tmc1ex1* were expressed than *mTmc1ex2*. Secondly, mTMC1 was translated from *mTmc1ex1* but not from *mTmc1ex2* in a heterologous expression system. Finally, analyses using site-directed mutagenesis revealed that the uORF and the weak Kozak sequence in *mTmc1ex2* prevented the translation of mTMC1 from *mTmc1ex2*. These results suggest that *mTmc1ex1* plays a main role in the expression of mTMC1 in the mouse cochlea, and therefore, *mTmc1ex1* should be the mRNA for mTMC1 hereafter.

## 1. Introduction

Transmembrane channel-like protein 1 (TMC1) forms the mechanoelectrical transduction (MET) channel, a type of mechanosensitive channel, at the tips of stereocilia of hair cells in the inner ear. When the stereocilia are tilted by sound wave or gravity, the MET channel opens and transduces these force into electrical signals by depolarizing membrane potentials [1]. Several mutations of *Tmc1* have been reported to cause hereditary deafness in mice and human [1]. Mice have been extensively used as an experimental animal in order to reveal how TMC1 contributes to the function of the MET channel [1].

**Figure 1:**
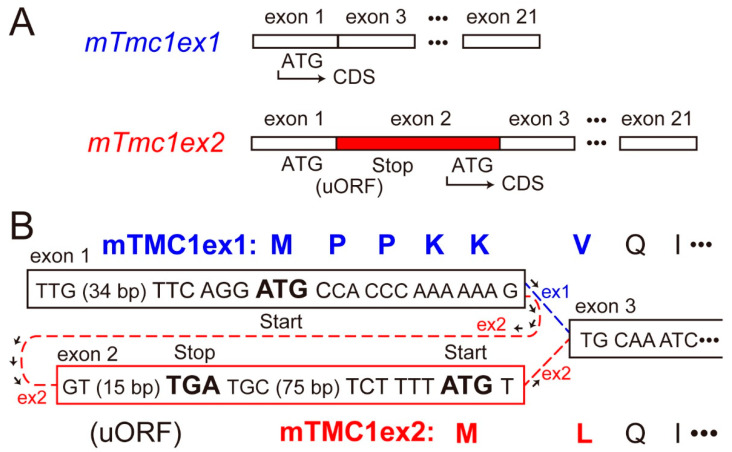
Differences in the splice variants of mouse *Tmc1* (*mTmc1*) and their translated proteins: (**A**) the exons which form mRNA of *mTmc1* splice variants, *mTmc1ex1* and *mTmc1ex2*. Exons 4–20 are omitted. The difference between *mTmc1ex1* and *mTmc1ex2* is the absence and presence of exon 2 in *mTmc1ex1* and *mTmc1ex2*, respectively. CDS: coding sequence. uORF: upstream open reading frame. (**B**) Nucleotide sequences around each start codon of *mTmc1ex1* and *mTmc1ex2* cDNAs (in squares) and translated amino acid sequences of mTMC1ex1 and mTMC1ex2 (capital letters at the top and the bottom, respectively): the sequence between the start codon for mTMC1ex1 in exon 1 and the stop codon (TGA) in exon 2 is uORF before the CDS of *mTmc1ex2*.

As transcripts of the mouse *Tmc1* (*mTmc1*) gene, two splice variants (*mTmc1ex1* and *mTmc1ex2*) were reported [2]. *mTmc1ex2* includes exon 2 containing its start codon, but *mTmc1ex1* excludes the exon 2 and uses AUG (ATG in the case of complementary DNA (cDNA)) in exon 1 as its start codon [2] (Figure 1A). As a result, the *N*-terminal amino acid residues of mTMC1ex1 and mTMC1ex2 are different (Figure 1B). Expression levels of *mTmc1ex1* and *mTmc1ex2* were similar to each other in either utricular maculae or cochleae at least within the first two weeks after birth in mice [2]. However, their expression levels in mature mice have not been evaluated as far as we know.

The cDNA sequence of *mTmc1* mRNA deposited in Genbank had been only *mTmc1ex2* (accession number: NM_028953.2). (Now, the *mTmc1ex1* sequence we deposited in Genbank is available (accession number: LC566569).) However, we questioned whether the mTMC1 protein is translated from *mTmc1ex2* for the following two reasons. first, the sequence around the start codon of *mTmc1ex2* (tctTttATGT) does not match the typical Kozak sequence (gccRccATGG, R = A/G), which is the consensus sequence for translation initiation [3]. Especially, purine nucleotide (R, adenine or guanine) at −3 position (where A of ATG is numbered +1) and guanine (G) at +4 position are important in the initiation of translation [3], but they are at variance in *mTmc1ex2*. On the other hand, the sequence around the start codon of *mTmc1ex1* (ttcAggATGC) contains a crucial A at the −3 position. Second, there is an upstream open reading frame (uORF) in exon 1, translation of which starts with ATG for *mTmc1ex1* (Figure 1A). Therefore, ribosome subunits are thought to dissociate and to be separated from the mRNA of *mTmc1ex2* after its stop codon of uORF unless the ATG for uORF is not recognized as the start codon (so called, “leaky scanning” [4]) or the ribosome reinitiates translation of mTMC1ex2 (so called, “ribosome re-initiation” [4]).

Therefore, in this study, we examined which splice variant was mainly expressed in the cochlea of mature mice and whether mTMC1 was translated from *mTmc1ex1* and *mTmc1ex2* using a heterologous expression system. Our results suggest that *mTmc1ex1* is the splice variant which functions as mRNA for mTMC1.

## 2. Results

### 2.1. Expression of mTmc1ex1 and mTmc1ex2 in the Cochlea of 5-Week-Old Mice

**Figure 2:**
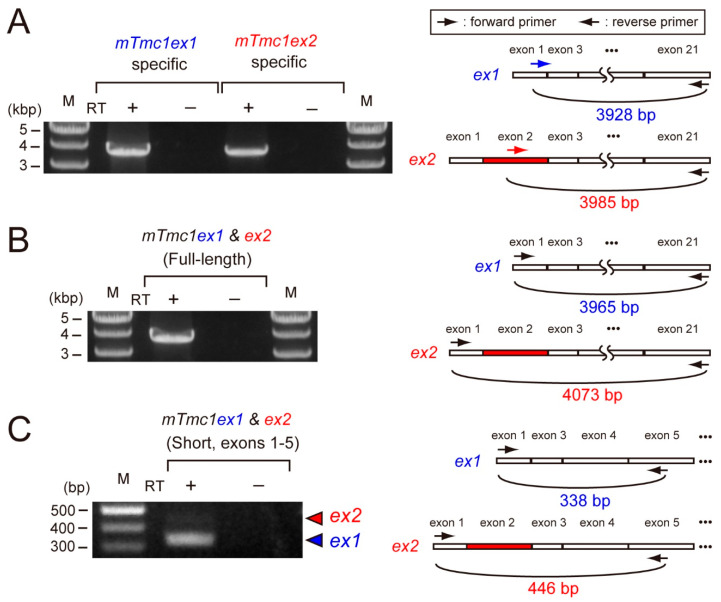
*mTmc1ex2* was expressed in the cochlea of 5-week-old mice, but the expression level of *mTmc1ex2* was lower than that of *mTmc1ex1:* (**A**) the result of RT-PCR using RNA extracted from the cochlea of a 5-week-old mouse and primer pairs with which either *mTmc1ex1* or *mTmc1ex2* can be amplified selectively (**left**). Positions of primers are shown as arrows in the illustrations of *mTmc1ex1* and *mTmc1ex2* (**right**). The forward primer for *mTmc1ex1* is an *mTmc1ex1*-specific primer as it spans exon 1 and exon 3 (**right upper**). The forward primer for *mTmc1ex2* is an *mTmc1ex2*-specific primer as it binds to exon 2 (**right lower**). M: marker. RT: reverse transcription. No amplification in the sample using mRNA as a template (RT−) indicates that the amplicon in the sample using cDNA as a template (RT+) was not due to contamination of genomic DNA. (**B**) The result of RT-PCR using a primer pair with which both full-length *mTmc1ex1* and full-length *mTmc1ex2* can be amplified: the expected sizes of amplicons of *mTmc1ex1* and *mTmc1ex2* are 3966 bp and 4073 bp, respectively. (**C**) The result of RT-PCR using a primer pair with which both *mTmc1ex1* and *mTmc1ex2* can be amplified as short fragments which are different in length (**left**): the expected sizes of amplicons of *mTmc1ex1* and *mTmc1ex2* are shown with a blue triangle (338 bp) and a red triangle (446 bp), respectively. The band for *mTmc1ex1* was obvious, but the one for *mTmc1ex2* was faint. The difference in the expected sizes of the amplicons is due to the length of exon 2 (**right**). Each RT-PCR reaction (**A**–**C**) was repeated three times using cDNA libraries produced independently from three mice, and similar results were obtained.

First, we examined the mRNA expressions of *mTmc1ex1* and *mTmc1ex2* in the cochlea of five-week-old mice. By using primer pairs which can amplify either *mTmc1ex1* or *mTmc1ex2* specifically, both *mTmc1ex1* and *mTmc1ex2* were amplified (Figure 2A). This result indicates that both variants were expressed at least as much as could be detected by RT-PCR. Next, in order to compare their expression levels, we used another primer pair which can amplify both *mTmc1ex1* and *mTmc1ex2* (expected sizes: 3966 bp and 4073 bp, respectively). The size of the amplicon appeared to be less than 4000 bp, indicating that the amplicon was composed of *mTmc1ex1* (Figure 2B). Cloning of the amplicon also indicated that almost all clones (28 out of 29 clones) did not contain exon 2 (i.e., They are not *mTmc1ex2*. See Supplementary Figure S1 (Supplementary Materials)). Furthermore, in order to make the difference in length of amplicons of variants more obvious, we used a primer pair which amplifies the short fragment spanning exon 2 (the expected sizes of *mTmc1ex1* and *mTmc1ex2* are 338 bp and 446 bp, respectively). With the primer pair, the band derived from *mTmc1ex1* was mainly amplified (the shorter band) but the band from *mTmc1ex2* (the longer band) was faint (Figure 2C). These results suggest that mainly *mTmc1ex1* is expressed in the cochlea of five-week-old mice.

### 2.2. mTMC1 Is Translated from mTmc1ex1 but Not from mTmc1ex2 due to the Presence of uORF and the Lack of Kozak Sequence

**Figure 3:**
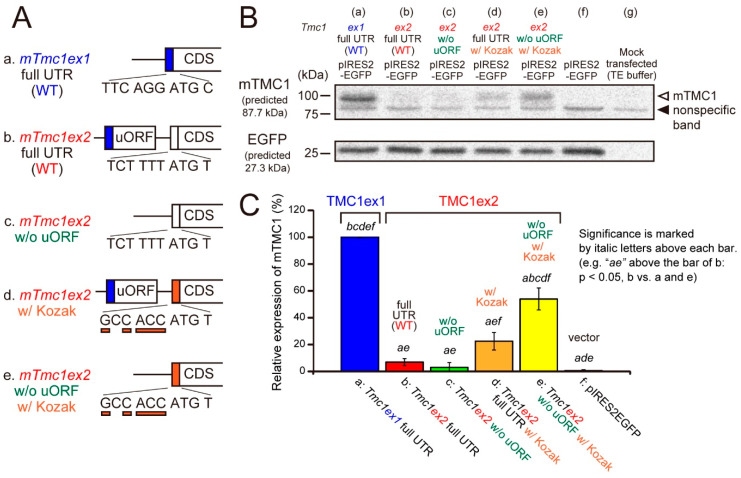
The mTMC1 protein was translated from *mTmc1ex1* but not from *mTmc1ex2* unless uORF was removed and the Kozak sequence was added. (**A**) cDNA structures of 5′ regions of *mTmc1ex1*, *mTmc1ex2*, and mutants of *mTmc1ex2*: (**a**) wild-type (WT) *mTmc1ex1* and (**b**) WT *mTmc1ex2*. (**c**) uORF was removed from the 5′ untranslated region (UTR) of *mTmc1ex2*. (**d**) The six nucleotides before the initiation codon of *mTmc1ex2* were mutated to the typical Kozak sequence (GCC ACC). (**e**) uORF was removed, and the Kozak sequence was added in *mTmc1ex2*. (**B**) A typical result of western blotting. mTMC1 was detected by an anti-TMC1 antibody (**upper**). The positions of mTMC1 and a nonspecific band were indicated by an open arrowhead and a filled arrowhead, respectively. The bands of the enhanced green fluorescent protein (EGFP) indicate similar levels of transfection of the vectors (**lower**). Lysates of empty vector-transfected cells (**f**) and mock transfected cells (**g**) were also loaded. Signals of the mock transfected cells were used as negative controls for the analysis of expression values. The expressions of nonspecific bands were not significantly different between each group (Supplementary Figure S3 (Supplementary Materials)). (**C**) Expression values of the mTMC1 protein normalized to that of the cells transfected with *mTmc1ex1* full UTR. Shown are means ±S.E. (*n* = 4). In order to reduce the differences in the transfection efficiencies, the signals for mTMC1 were divided by the signals for EGFP before normalizations. Italic letters over the bars indicate the presence of significant differences from the group indicated by the italic letters (*p* < 0.05, Tukey’s test).

Next, we examined whether the mTMC1 protein can be translated from *mTmc1ex1* and *mTmc1ex2* using a heterologous expression system and Western blot analyses. cDNAs of *mTmc1ex1* or *mTmc1ex2* were subcloned into a bicistronic expression vector (pIRES2-EGFP), which expresses both a protein encoded by the inserted cDNA and an enhanced green fluorescent protein (EGFP). By detecting EGFP expression, we can confirm the success of transfection. When the vector containing *mTmc1ex1* was transfected into HEK293T cells, the mTMC1 protein was detected by western blotting (Figure 3A, *mTmc1ex1* full untranslated region (UTR) (wild type, WT)). On the other hand, when the vector containing *mTmc1ex2* was transfected, mTMC1 was not detected (Figure 3B, *mTmc1ex2* full UTR (WT) with no significant difference from a group transfected with an empty vector (f, pIRES-EGFP)). Similar results were obtained even when another expression vector was used (pcDNA3.1(+), Supplementary Figure S2 (Supplementary Materials)). These results indicate that *mTmc1ex1* is a splice variant from which mTMC1 is translated and that *mTmc1ex2* does not function as an mRNA for mTMC1.

Furthermore, we tried to uncover the reasons for the negligible translation of mTMC1 from *mTmc1ex2*. First, a removal of uORF from 5′ UTR of *mTmc1ex2* did not increase the expression of mTMC1 (Figure 3B(c)). Second, mutations of six nucleotides before the initiation codon of *mTmc1ex2* to the typical Kozak sequence (GCC ACC) slightly increased the expression of mTMC1 (Figure 3B(d)), but there was no significant difference from WT *mTmc1ex2* (b). A combination of the removal of uORF and the addition of the typical Kozak sequence significantly increased the mTMC1 expression (Figure 3B(e) vs. (b–d) and (f)) though the expression value was also significantly smaller than WT *mTmc1ex1* (vs. (a)). Similar results were obtained using pcDNA3.1(+) also (Supplementary Figure S2 (Supplementary Materials)). These results indicate that negligible translation of mTMC1 from *mTmc1ex2* is, first, due to the weak Kozak sequence of *mTmc1ex2* and, second, due to the presence of uORF in the 5′ UTR of *mTmc1ex2*.

### 2.3. Both the Heterologously Expressed mTMC1ex1 and mTMC1ex2 Were Retained in Cytoplasm

**Figure 4:**
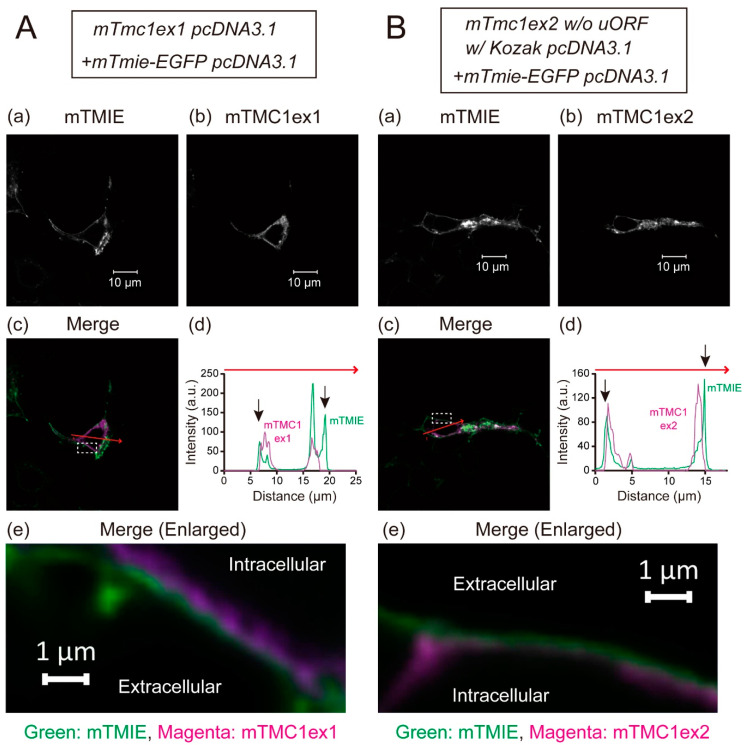
Representative images showing distributions of heterologously expressed mTMC1ex1 (**A**) and mTMC1ex2 (**B**) in HEK293T cells: (**a**) the signals of EGFP-tagged mouse transmembrane inner ear (mTMIE), which was co-expressed, and (**b**) the signals of mTMC1ex1 and mTMC1ex2, which were expressed using the WT *mTmc1ex1* and the mutant *mTmc1ex2* (*w*/*o* uORF and w/Kozak sequence) in the pcDNA3.1 vector, respectively. (**c**) In the merged images, the signals of mTMIE are shown in green and the signals of mTMC1 are shown in magenta. (**d**) Signal intensities were measured along the randomly chosen red arrows on the merged images. The black arrows indicate the positions of mTMIE at the periphery of the cells. The signals of mTMIE were observed outside of those of mTMC1ex1 and mTMC1ex2. (**e**) Enlarged merged images of the area squared with a white dotted line in (**c**): The green signals of mTMIE outlined the magenta signals of mTMC1.

The heterologously expressed TMC1 is known not to be targeted to the plasma membrane and to be retained in cytoplasm [5]. That disables functional analyses of mTMC1 in heterologous expression systems. In this study, mTMC1ex1 is suggested to be a protein encoded by the *mTmc1* gene. Although the difference between mTMC1ex1 and mTMC1ex2 is only a few amino acid residues at their *N*-terminus (Figure 1B), we examined whether mTMC1ex1 can be targeted to the plasma membrane. EGFP-tagged transmembrane inner ear (TMIE), a component of the MET channel but not a direct interacting protein of TMC1 [6], was co-expressed in order to make the plasma membrane visible as TMIE is targeted at the plasma membrane [6]. Confocal microscopy revealed that heterologously expressed mTMC1ex1 was mainly localized in the intracellular space of the cell as well as mTMC1ex2 (Figure 4A(b),B(b)). At least most signals of both mTMC1ex1 and mTMC1ex2 were not colocalized with mouse TMIE (mTMIE) at the periphery of the cells (Figure 4A,B(c–e)), suggesting that neither mTMC1ex1 nor mTMC1ex2 accumulated in the plasma membrane. Therefore, their different *N*-terminal amino acid residues were less likely to be a determinant of their plasma membrane targeting.

## 3. Discussion

Our results strongly suggest that *mTmc1ex1* is the splice variant which functions as mRNA for mTMC1 due to the following observations. First, the results of RT-PCR and cDNA cloning suggest that *mTmc1ex1* is mainly expressed in mature cochlea. Second, results of western blotting in a heterologous expression system indicate that mTMC1 is translated from *mTmc1ex1* but is not translated from *mTmc1ex2* because of the presence of uORF and the absence of the typical Kozak sequence in its 5′ UTR. We cannot completely deny the possibility that the presence of cDNA encoded by exon 2 inhibited RT-PCR reactions. However, even if *mTmc1ex2* mRNA was actually expressed in the cochlea more than suggested by our results, the translation of mTMC1 from *mTmc1ex2* would be negligible because of the sequence of its 5′ UTR. Additionally, as another supporting evidence, a known human *TMC1* cDNA (*hTMC1*, Genbank accession number: NM_138691.3) is equivalent to *mTmc1ex1*, and its *N*-terminal amino acid residues (MSPKKVQI…, NP_619636.2) resembles that of mTMC1ex1 (MPPKKVQI…) rather than that of mTMC1ex2 (MLQI…). Moreover, a splice variant of *hTMC1* equivalent to *mTmc1ex2* has not been reported as far as we know. Therefore, this information on *hTMC1* also supports that *mTmc1ex1* is the mRNA for mTMC1.

One significance of our findings is that they show that *mTmc1ex1* should be used as the mRNA of the *mTmc1* gene. If a primer is designed from the sequence of exon 2, the primer detects only *mTmc1ex2*. Therefore, the RT-PCR results using the primer will not give correct information on the expression levels of mRNA which translates mTMC1. Moreover, even if primers for RT-PCR and probes for Northern blotting are designed from the common sequence of *mTmc1ex1* and *mTmc1ex2* (the sequence other than exon 2), they detect both *mTmc1ex1* and *mTmc1ex2*. The expression of *mTmc1* has been reported in cochlea, eye, testis, and others in many cases without discriminating *mTmc1ex1* and *mTmc1ex2* [7,8]. If the detected *mTmc1* mRNAs are mainly *mTmc1ex2*, the actual expression level of the mTMC1 protein will be low. Therefore, when *mTmc1* mRNA is detected, it should be done in a *mTmc1ex1*-specific manner.

Moreover, although the difference in amino acid sequence between mTMC1ex1 and mTMC1ex2 is only a few amino acid residues, the mTMC1 protein which is composed of the genuine amino acid sequence (i.e., mTMC1ex1) should be used for analyses in heterologous expression systems. In heterologous expression systems, in order to increase the expression level, the Kozak sequence is sometimes added to the sequence before the start codon, as done in a research on mTMC1 using *mTmc1ex2* [5]. Such an artificial modification is useful and thought to be accepted because the translated amino acid sequence is not changed. However, as shown in our study, it may cause researchers to conduct experiments using proteins which are not actually translated very much.

The difference in a few amino acid residues at the *N*-terminus between mTMC1ex1 and mTMC1ex2 might not cause any functional differences between them. At least, when they were heterologously expressed, their localizations (i.e., retentions in cytosol) were not changed. However, it was recently reported that co-expression of KCNQ1, a K^+^ channel, rescued hTMC1 plasma membrane targeting [9] although KCNQ1 is not thought to be an endogenous interacting protein of TMC1 in hair cells in the inner ear because of the lack of endogenous expression [9]. If the interaction with KCNQ1 is mediated by the *N*-terminus of TMC1, the difference between mTMC1ex1 and mTMC1ex2 may affect their interaction with KCNQ1. Additionally, even when KCNQ1 was co-expressed, channel activities of hTMC1 were not detected, suggesting requirements of other components for the formation of a functional channel by TMC1 [9]. When the functional analyses of TMC1 in heterologous expression systems become possible, the functional differences between mTMC1ex1 and mTMC1ex2 might be revealed.

Although our results suggest that mTMC1 is not translated from *mTmc1ex2*, it was reported that *mTmc1ex2* was expressed at the level similar to *mTmc1ex1* in the cochlea at least within the first two weeks after birth in mice [2]. What is the meaning of the presence of *mTmc1ex2*?

First, *mTmc1ex2* might be expressed in order to reduce the expression level of mTMC1 protein in the immature cochlea. It was reported that, in the first week after birth, *mTmc2* (an orthologue of *mTmc1*) was mainly expressed in the cochlea and *mTmc1* expression was low [2,10]. After that, the expression of *mTmc2* decreased but *mTmc1* expression increased [2,10] although the meaning of this switch from mTMC2 to mTMC1 during development has not been clarified. The expression of *mTmc1ex2* as a splice variant of *mTmc1* in the immature cochlea may result in the lower expression of mTMC1 protein that might be useful for mTMC2 to play a major role in mechanoelectrical transduction instead of mTMC1. On the other hand, our present study indicates that *mTmc1ex1* is the major splice variant of *mTmc1* in the cochlea of 5-week-old mice. Therefore, the dominant expression of *mTmc1ex1* may be advantageous for sufficient expression of mTMC1 in the mature cochlea.

Second, the peptide encoded by the uORF in *mTmc1ex2* might have physiological functions. The uORF contains the Kozak sequence, which is identical to that of *mTmc1ex1*. Therefore, the peptide encoded by the uORF is supposed to be translated from *mTmc1ex2* mRNA. The amino acid sequence of the peptide is MPPKKGVSGHF. As the peptide is very short and its first five amino acid residues out of 11 are identical to the *N*-terminus of mTMC1ex1, it will be difficult to specifically detect the short peptide in the cells which express mTMC1 using antibodies. However, there is a possibility that the short peptide has some functional roles as it has been reported that such short peptides encoded by uORF in some mRNAs were translated and showed physiological functions such as translational regulations [4]. Further research will be required to understand the meaning of the presence of *mTmc1ex2*.

In conclusion, the present study suggested that *mTmc1ex1* plays a main role in the expression of mTMC1 protein in mouse cochlea, and therefore, *mTmc1ex1* should be treated as the mRNA for mTMC1 hereafter. Furthermore, our findings recommend that researchers pay more attention to 5′ UTR sequences (uORF and Kozak sequence) in mRNAs of proteins in which they are interested in order to avoid conducting research on proteins which are not actually translated.

## 4. Materials and Methods

### 4.1. Animal Ethics Approval

All animal experiments were performed in accordance with guidelines from and protocols approved by the Institutional Animal Care and Use Committee, Graduate School of Veterinary Medicine, Hokkaido University (Protocol number 14-0060, 13/5/2014).

### 4.2. RT-PCR

RNAs were extracted from cochlea of male C57BL/6N mice (five weeks old, Crea Japan, Tokyo, Japan) using NucleoSpin RNA II (Takara Bio, Otsu, Japan). cDNA was synthesized using PrimeScript II Reverse Transcriptase (Takara Bio). In order to evaluate the expression levels of *mTmc1ex1* and *mTmc1ex2*, PCR was conducted using a high-fidelity polymerase (PrimeSTAR GXL, Takara Bio), and the primers are listed in Table 1. The template volume was 750 ng as RNA. The PCR cycle number was 35 cycles. The primers named “mTmc1 5′UTR F” and “mTmc1 3′UTR R” were designed in order to amplify the whole sequence of the deposited *mTmc1ex2* cDNA (NM_028953.2). Simultaneously, whole *mTmc1ex1* cDNA also can be amplified with these primers. The design of the other primers was explained in the Results section. The PCR products were analyzed by agarose gel electrophoresis followed by ethidium bromide staining.

### 4.3. Molecular Cloning and Site-Directed Mutagenesis

The full-length of *mTmc1* cDNA was amplified by PCR using PrimeSTAR GXL and the primers “mTmc1 5′UTR F” and “mTmc1 3′UTR R” (shown in Table 1). The PCR product (the amplified *mTmc1* cDNA) was cloned in pGEM-T Easy Vector (Promega, Madison, WI, USA), and four clones were sequenced. The presence or absence of exon 2 in these clones were examined by PCR using a primer pair: “mTmc1 5′UTR F” and “mTmc1 R”. The *mTmc1ex1* cDNA which contained no PCR errors was subcloned in a bicistronic expression vector, pIRES2-EGFP vector (Clontech Laboratories, Mountainview, CA, USA) or pcDNA3.1(+) vector (Takara Bio). In the case of *mTmc1ex2*, there was a deletion of 41 nucleotides in CDS in the only clone obtained (#20 in Supplementary Figure S1 (Supplementary Materials)). Therefore, when *mTmc1ex2* cDNA was subcloned into the expression vectors, part of the deletion was substituted by the sequence obtained as *mTmc1ex1* so that the full-length WT *mTmc1ex2*, which is the same as the whole sequence of *mTmc1ex2* deposited in Genbank (NM_028953.2), was artificially constructed. The mutants of *mTmc1ex2* were made by site-directed mutagenesis using the PrimeSTAR Mutagenesis Basal kit (Takara Bio). Mutations were verified by sequencing.

### 4.4. Cell Culture and Transfection

HEK 293T cells were obtained from the RIKEN BioResource Research Center through the National Bio-Resource Project of the MEXT, Japan. HEK 293T cells were cultured in Dulbecco’s modified Eagle’s medium (Sigma-aldrich, St. Louis, MO, USA) supplemented with 10% fetal bovine serum (Thermo Fisher Scientific, Waltham, MA, USA) and penicillin/streptomycin (100 U/mL and 100 μg/mL, respectively, FUJIFILM Wako Pure Chemical Corporation, Osaka, Japan) at 37 °C in a 5% CO_2_ incubator. Cells were transiently transfected with plasmids using TransIT-293 Transfection Reagent (Takara Bio). Thirty to forty-eight hours after the transfection, the cells were used for experiments.

### 4.5. Western Blotting

Western blotting was conducted as described previously [11] with some modifications. Cells were lysed in a lysis buffer (150 mM NaCl, 2 mM ethylenediaminetetraacetic acid (EDTA)·2Na, 10 mM 4-(2-hydroxyethyl)-1-piperazineethanesulfonic acid (HEPES), 1% Triton X-100, and pH = 7.4) by sonication. The lysates were centrifuged at 14,000× *g* for 10 min at 4 °C. The supernatants were mixed with equal volume of urea sample buffer (8 M urea, 5% SDS, 0.1% bromophenol blue, 0.2 M Tris·Cl (pH = 6.8), and 0.1 M dithiothreitol (DTT) [12]) and heated at 37 °C for 10 min. This treatment dissociated mTMC1 protein aggregates and increased the detection of monomeric mTMC1 proteins. Proteins (10 μg each) were separated by SDS-PAGE and transferred to a polyvinylidene difluoride (PVDF) membrane (Bio-Rad Laboratories, Hercules, CA, USA). After blocking with skim milk, the membrane was incubated with a commercially available anti-mTMC1 rabbit antibody (ab199949, abcam, Cambridge, UK) or an anti- green fluorescent protein (GFP) rabbit antibody (#598, MBL, Nagoya, Japan) and an horseradish peroxidase (HRP)-conjugated anti-rabbit IgG secondary donkey antibody (GE healthcare, Chicago, IL, USA). The chemiluminescent signals were produced by using ECL prime (GE healthcare) and detected using a digital camera (Canon, Tokyo, Japan). The chemicals used were obtained from Sigma-aldrich (St. Louis, MO, USA), Kanto Chemical (Tokyo, Japan), nacalai tesque (Kyoto, Japan), and FUJIFILM Wako Pure Chemical Corporation (Osaka, Japan).

### 4.6. Immunostaining

Cells which were co-transfected with *mTmie*-EGFP pcDNA3.1 (cloned from cDNA of cochlea) and *mTmc1ex1* pcDNA3.1 or *mTmc1ex2* *w*/*o* uORF w/Kozak pcDNA3.1 on coverslips were fixed with 4% paraformaldehyde and permeabilized with 0.1% TritonX-100. Nonspecific staining was blocked by incubations with image-iT FX signal enhancer (Thermo Fisher Scientific) and with PBS containing 2% bovine serum albumin and 5% normal goat serum. The primary antibody was a custom-made anti-mTMC1 polyclonal rabbit antibody against mTMC1 (antigen: DEETRKAREKERRRRLRRG, 57th–75th amino acid residues of mTMC1ex1 (53rd–71st of mTMC1ex2), Sigma-Aldrich). The secondary antibody was Alexa 555-conjugated goat anti-rabbit IgG F(ab’)2 fragment (Thermo Fisher Scientific). After washings, the coverslips were mounted with Prolong Diamond (Thermo Fisher Scientific). Fluorescence was observed using a confocal microscope (LSM 700, Carl Zeiss, Jena, Germany).

## Figures and Tables

**Table 1:** Sequences of the primers used.

Figures	Amplicon	Direction	Name	Nucleotide Sequence (5′ to 3′)
Figure 2A	*mTmc1ex1*	Forward	mTmc1ex1_F	TTCAGGATGCCACCCAAAAAAGTGC
		Reverse	mTmc1 3′UTR R	GAAATCAACACGATCTTTATTTGCTGC
	*mTmc1ex2*	Forward	mTmc1ex2_F	GCCTGTCTTCCTCTTAGCTCCTGTC
		Reverse	mTmc1 3′UTR R	GAAATCAACACGATCTTTATTTGCTGC
Figure 2B	*mTmc1ex1* & *mTmc1ex2*	Forward	mTmc1 5′UTR F	TTGCAATTCCTGATTAGAGACATTCTG
		Reverse	mTmc1 3′UTR R	GAAATCAACACGATCTTTATTTGCTGC
Figure 2C	*mTmc1ex1* & *mTmc1ex2*	Forward	mTmc1 5′UTR F	TTGCAATTCCTGATTAGAGACATTCTG
		Reverse	mTmc1 R	GTCTATTCTCATCGAGCAGTGCTTTTAAC

## Supplementary Materials

The following are available online at https://www.mdpi.com/1422-0067/21/18/6465/s1. Supplementary Materials can be found in an additional file.

## Abbreviations

TMC1	Transmembrane channel-like protein 1
*Tmc1*	Gene, mRNA, or cDNA for TMC1
mTMC1	Mouse TMC1
MET	Mechanoelectrical transduction
uORF	Upstream open reading frame
cDNA	Complementary DNA
CDS	Coding sequence
EGFP	Enhanced green fluorescent protein
RT	Reverse transcription
M	Marker
UTR	Untranslated region
WT	Wild type
hTMC1	Human TMC1
