# Supplementary material for: A Mechanosensitive Channel, Mouse Transmembrane Channel-Like Protein 1 (mTMC1) Is Translated from a Splice Variant mTmc1ex1 but Not from the Other Variant mTmc1ex2
Source: Int J Mol Sci. 2020 Sep 4;21(18):6465. doi: 10.3390/ijms21186465 (PMC7554986; doi:10.3390/ijms21186465)
Supplement: Supplementary file 1 [file ijms-21-06465-s001.pdf]

International Journal of Molecular Sciences

MDPI

Supplementary Materials for "A mechanosensitive channel, mouse transmembrane channel-like protein 1 (mTMC1) is translated from a splice variant *mTmc1ex1* but not from the other variant *mTmc1ex2*", Yamaguchi et. al.

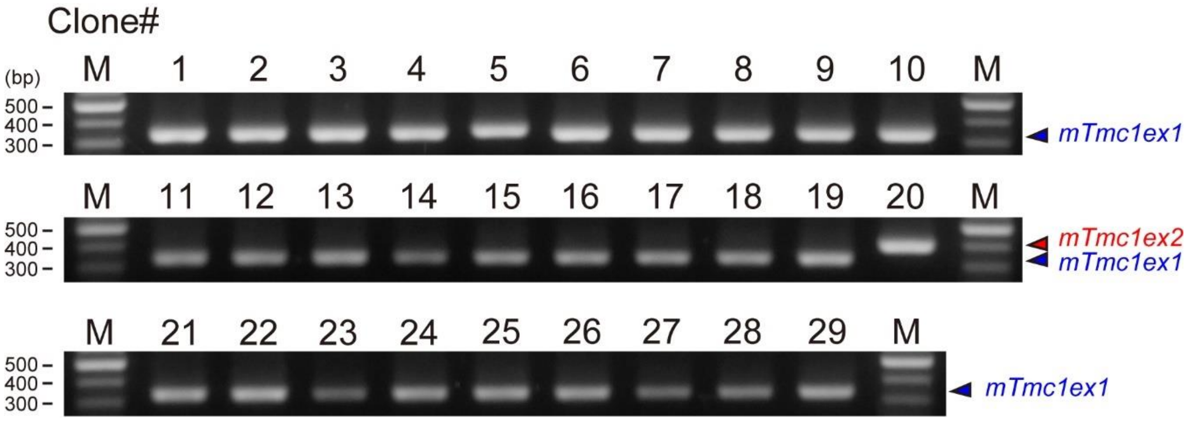

Supplementary Figure S1. PCR analyses using plasmids (containing full-length *mTmc1ex1 or mTmc1ex2*) as templates in order to determine the absence or presence of exon 2. The used primer pair is the same with the one used in Figure 2C. The expected sizes of amplicons of *mTmc1ex1* and *mTmc1ex2* are shown with a blue triangle (338 bp) and a red triangle (446 bp), respectively. Inserted cDNAs of clones #3, 5, 10, and 20 were sequenced. Clones #3, 5, and 10 were confirmed to be *mTmc1ex1*. Clone #20 contained exon 2 but there was a deletion of 41 nucleotides in CDS. Therefore, when the WT *mTmc1ex2* was constructed in an expression vector, the deleted region was filled using the sequence of *mTmc1ex1* as described in the Materials and Methods section.

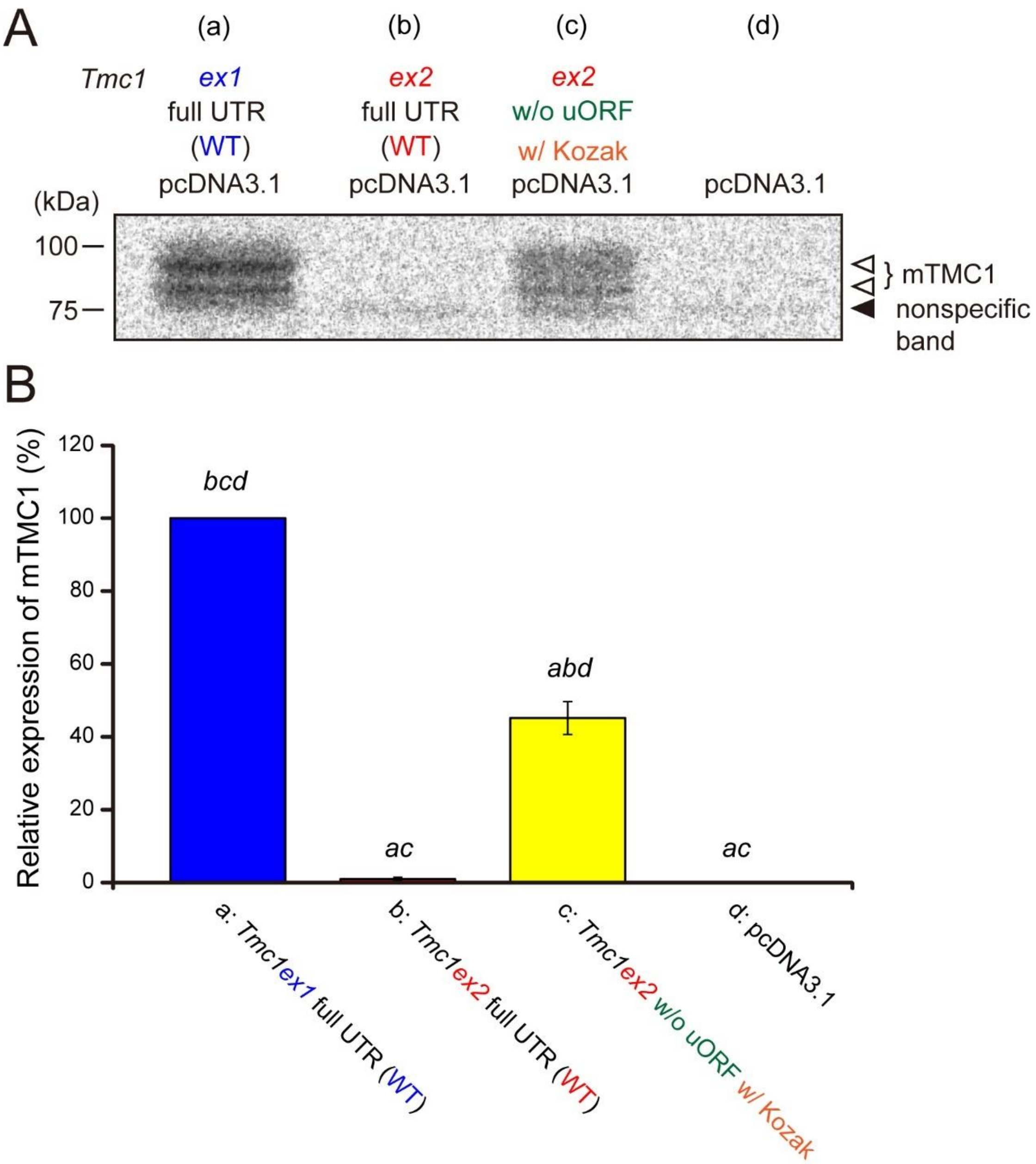

Supplementary Figure S2. mTMC1 protein was translated from *mTmc1ex1* but not from *mTmc1ex2* unless uORF was removed and the Kozak sequence was added even with another expression vector pcDNA3.1(+). (A) A typical result of a western blotting. mTMC1 was detected by an anti-TMC1 antibody. The positions of mTMC1 and a nonspecific band were indicated by open arrowheads and a filled arrowhead, respectively. The lower mTMC1 band was faintly visible also in Figure 2B. However, although the reason is unknown, the lower band of mTMC1 was more obvious when the pcDNA3.1(+) vector was used. The double bands of mTMC1 were also observed elsewhere [5] and the reason for them remains elusive. (a) WT *mTmc1ex1*. (b) WT *mTmc1ex2*. (c) A mutant of *mTmc1ex2* (uORF was removed and Kozak sequence was added). (d) An empty vector (pcDNA3.1). Signals of the empty-vector transfected cells were used as negative controls for the analysis of expression values. (B) Expression values of mTMC1 protein normalized to that of the cells transfected with WT *Tmc1ex1*. Shown are means ±S.E. (n = 3). Italic letters over the bars indicate the presence of significant differences from the group indicated by the italic letters ($p < 0.05$, Tukey's test).

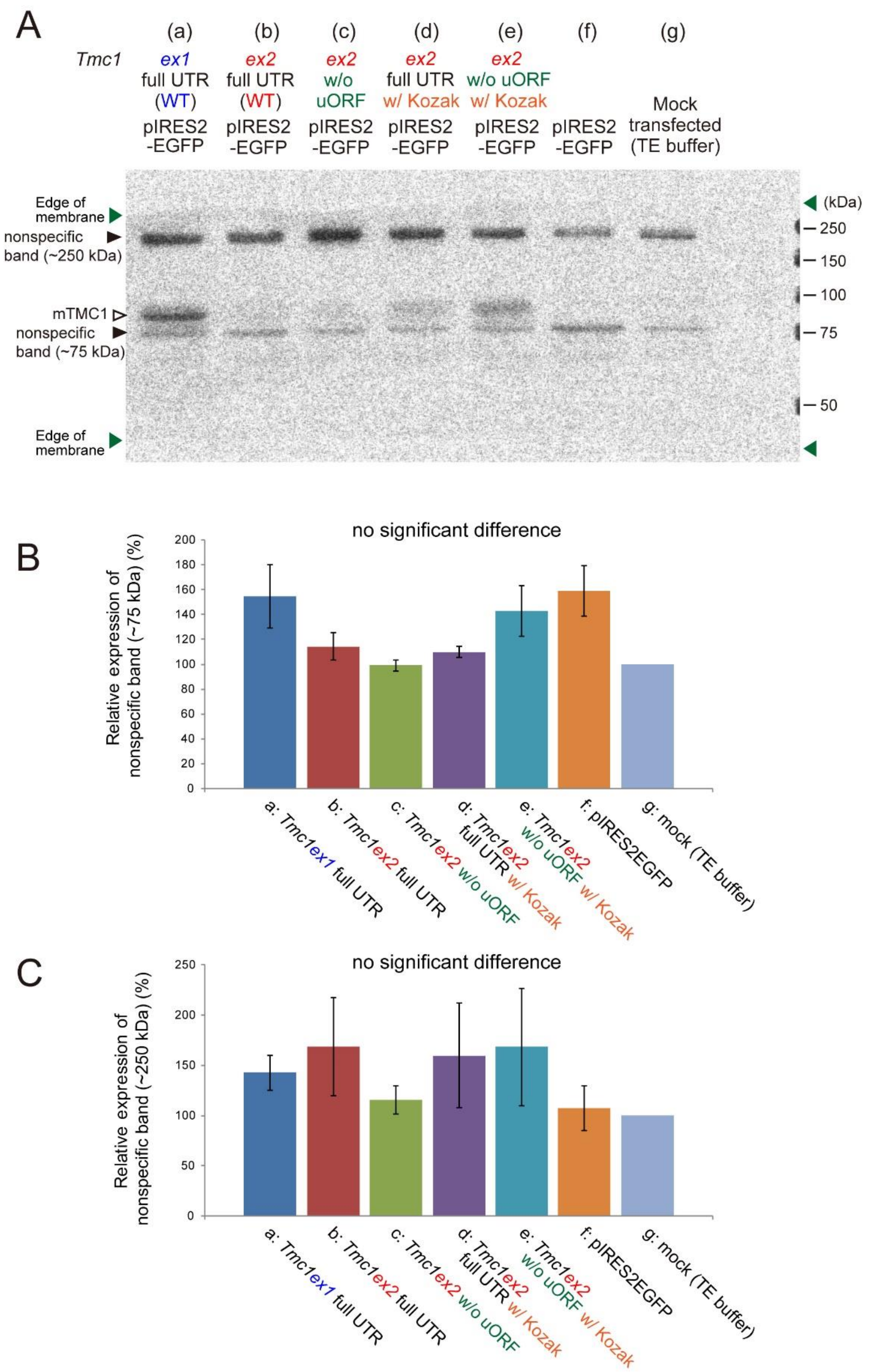

Supplementary Figure S3. Expressions of non-specific bands were not significantly different between each group. (A) The original picture of the full-size membrane which is shown in Figure 2B. Green arrowheads indicate the edges of the membrane. The positions of mTMC1 and nonspecific bands are indicated by the open arrowhead and the filled arrowheads, respectively. (B) Expression values of non-specific bands around 75 kDa, normalized to that of the mock transfected cells. Shown are means ±S.E. (n = 4). There is no significant difference ($p > 0.05$, Tukey's test). (C) Expression values of non-specific bands around 250 kDa, normalized to that of the mock transfected cells. Shown are means ±S.E. (n = 3). There is no significant difference ($p > 0.05$, Tukey's test).
